# Newborn screening for Tyrosinemia type 1 using succinylacetone – a systematic review of test accuracy

**DOI:** 10.1186/s13023-017-0599-z

**Published:** 2017-03-09

**Authors:** Chris Stinton, Julia Geppert, Karoline Freeman, Aileen Clarke, Samantha Johnson, Hannah Fraser, Paul Sutcliffe, Sian Taylor-Phillips

**Affiliations:** 1 Warwick Medical School, University of Warwick, Coventry, CV4 7AL England; 2 Warwick Library, University of Warwick, Coventry, CV4 7AL England

**Keywords:** Systematic review, Tyrosinemia, Test accuracy, Succinylacetone, Inborn errors of metabolism, Newborn blood spot screening, Tandem mass spectrometry

## Abstract

**Background:**

Tyrosinemia type 1 is an autosomal recessive disorder of amino acid metabolism. Without treatment, death in childhood is common. Treatment with nitisinone and dietary restrictions are associated with improved outcomes; some studies suggest better outcomes when treatment begins at an asymptomatic stage. Newborn screening allows for earlier identification, but there is uncertainty regarding the test accuracy of the current method: succinylacetone measurement in dried blood spots using tandem mass spectrometry.

**Methods:**

We conducted a systematic review of literature published up to January 2016. Two reviewers independently assessed titles, abstracts, full texts, and conducted quality appraisals. A single reviewer extracted data, which was checked by a second reviewer.

**Results:**

Ten studies provided test accuracy data: five studies reporting screening experiences and five case–control studies. Sensitivity (29 cases in total) and specificity (34,403 controls in total) were 100% in the case–control studies, but could not be calculated in the studies reporting screening experiences due to a lack of follow-up of screen-negative babies. Positive predictive values in the screening experience studies ranged from 66.7% (2 true positive cases, 1 false positive case from ~500,000 people screened) to 100% (8 true positive cases from 856,671 people screened); negative predictive values could not be calculated. Positive and negative predictive values cannot be calculated from case–control studies.

**Conclusions:**

Screening for Tyrosinemia type 1 using tandem mass spectrometry measurement of succinylacetone from dried blood spots appears to be promising. Confirmation of test accuracy data should be obtained from studies that include a two-year follow-up of individuals who screen negative.

**Electronic supplementary material:**

The online version of this article (doi:10.1186/s13023-017-0599-z) contains supplementary material, which is available to authorized users.

## Background

Tyrosinemia type 1 (TYR1), also known as fumarylacetoacetase deficiency (Enzyme Commission Number 3.7.1.2), is an autosomal recessive disorder of amino acid metabolism. It is caused by a deficiency in the activity of fumarylacetoacetic hydrolase, the final enzyme in the tyrosine degradation pathway, which leads to a toxic build-up of fumarylacetoacetate, maleylacetoacetate, and succinylacetone (SUAC) [1]. TYR1 is characterised by progressive liver, kidney, and neurological disease [2]. Acute (presenting before six months of age), sub-acute (presenting between six and 12 months) and chronic (presenting after one year) forms of the disease have been described [2]. Without treatment, the prognosis for individuals with TYR1 is poor, with high levels of death during childhood due to liver failure, recurrent bleeding, hepatocellular carcinoma, and porphyria-like syndrome with respiratory failure [3]. However, treatment with nitisinone and dietary restrictions are associated with reductions in morbidity and mortality [4–6]; liver transplantation is indicated if these treatment fail or if hepatocellular carcinoma develops [2]. The incidence of TYR1 is estimated to be approximately 1:100,000 live births, but reported values range from 1:1,846 [7] to 1:781,144 live births [8]. The incidence of TYR1 is higher in Quebec, Canada, possibly due to a founder effect for Tyrosinemia and high gene frequency [7], and in Asian children in the West Midlands of the UK [9], and in North Africa and the Middle East [10], possibly due to parental consanguinity [9].

Screening for TYR1 amongst newborn babies is conducted in many countries around the world. While tyrosine levels have been used as the primary screening marker for TYR1, it is not consistently raised in individuals who have TYR1 [11], and it can be elevated in individuals with other conditions and in unaffected babies [12, 13]. In 2004, Allard and colleagues developed an alternative method to screen for TYR1 using tandem mass spectrometry (MS/MS) to determine SUAC in dried blood spots (DBS) [14]. A rapid review of literature published up to 2012 reported that “Screening programmes using succinylacetone as a marker have reported 100% sensitivity and 100% specificity. However, other studies have reported the identification of false positives.” [15]. The aim of the current review was to examine the range of test accuracy indicators (sensitivity, specificity, and predictive values) of succinylacetone measurement in DBS using MS/MS for TYR1 screening using full systematic review methods.

## Methods

### Search strategy

We conducted searches in the following electronic databases: Medline, Medline In-Process & Other Non-Indexed Citations, Embase, Web of Science (All Databases), and the Cochrane Library. We searched using text word and MeSH terms relating to “Tyrosinemia type 1 OR inborn errors of metabolism”, AND “succinylacetone OR DBS OR (tandem mass spectrometry AND neonatal screening)”. Full details of the search strategy are provided in Additional file 1: supplement 1. The search was conducted on 26^th^ January 2016. We examined reference lists of included studies and previous reviews. Experts in the field and organisations were contacted for studies not in the public domain.

### Eligibility criteria

We included English language journal articles which investigated screening for TYR1 by MS/MS analysis of SUAC from DBS in newborns. The reference standard was urine testing for SUAC, clinical detection of TYR1 or two-year follow-up. Outcomes included were any reported test accuracy measures from cross-sectional studies, case–control studies, or studies reporting screening experiences. We excluded non-human studies, papers not available in English, letters, editorials, communications, grey literature, conference abstracts, and studies published before 2004 (the year the first paper was published on SUAC measurement in DBS using MS/MS for TYR1) from our review.

### Screening and data extraction

Screening of titles and abstracts of all retrieved records, and subsequently of full texts, was undertaken independently by two reviewers. Data extraction was performed by a single reviewer, with all data extraction forms checked by a second reviewer. Disagreements were resolved by discussion between the two reviewers or further discussion with a third reviewer, leading to a consensus on inclusion/exclusion.

### Quality appraisal

Quality of included studies was assessed independently by two reviewers using the Quality Assessment Tool for Diagnostic Accuracy Studies 2 [QUADAS-2; [16]] which was tailored to the research as recommended. Tailoring of the QUADAS-2 tool included adding a topic-specific signalling question and defining appropriate reference standards and cut-offs for participant exclusions, as well as guidance on how many positive signalling questions are required for an overall positive rating in terms of bias and applicability concerns. (See Additional file 1: supplement 2 for signalling questions and Additional file 1: supplement 3 for guidance notes). Disagreements were resolved by discussion between the two reviewers or through discussion with a third reviewer, leading to a consensus on study quality.

### Data summary and synthesis

Meta-analysis was not possible due to incomplete 2x2 tables and heterogeneity in study design. Therefore, a narrative synthesis of results is provided.

## Results

### Searching, sifting, and sorting

**Fig. 1:**
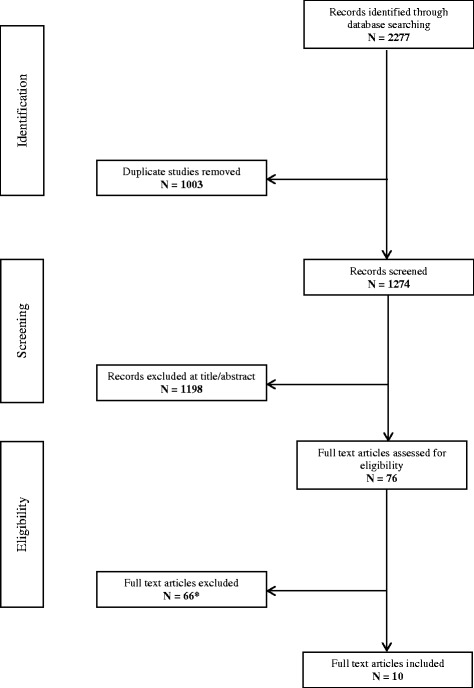
PRISMA flow diagram of records through the systematic review. *See Additional file 1: supplement 4 for list of excluded studies with reasons

One thousand two hundred and seventy-five unique records were identified. Seventy six were retained after sifting titles and abstracts. Assessment of full text papers against inclusion/exclusion criteria resulted in ten studies being included in our review; five studies reporting screening experiences [12, 17–20] and five retrospective case–control studies using stored samples from known TYR1 cases [14, 21–24]. All ten papers were identified through electronic database searches. Full details regarding the numbers of studies retained and excluded at each stage of the review is provided in Fig. 1. A list of excluded studies (with reasons) is given in Additional file 1: supplement 4.

### Quality appraisal

**Fig. 2:**
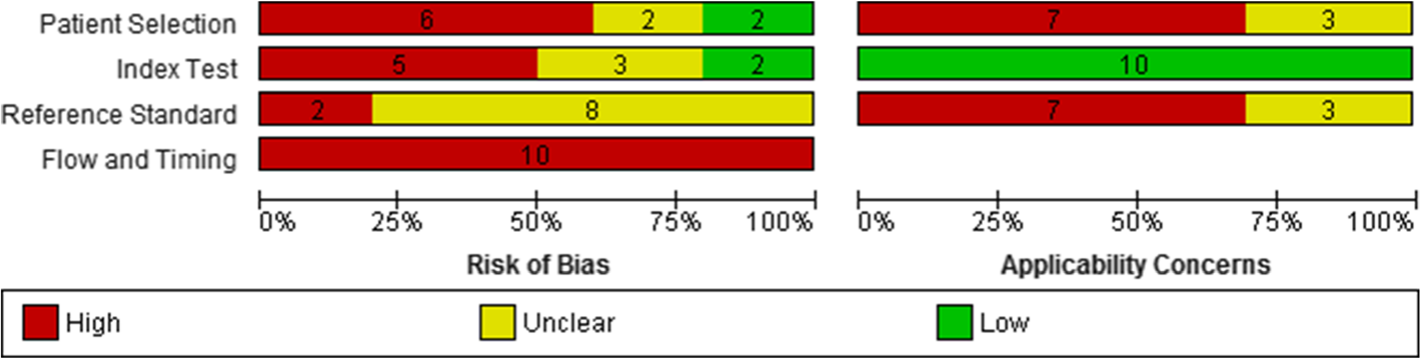
Risk of bias and applicability concerns graph: review authors’ judgements about each domain presented as percentages across included studies

The overall risk of bias and applicability concerns of the included studies are provided in Fig. 2. A summary of the methodological quality for each of the included studies is given in Additional file 1: supplement 5. Risk of bias was considered high in two or more domains in six of ten studies (60%) and in one domain in the remaining four studies (40%). No study was judged as low or unclear risk of bias in all four domains. In the patient selection domain, all five case–control studies [14, 21–24] were considered to be at a high risk of bias. One study reporting a screening experience [20] was considered to be at a high risk of bias as the study population included screening samples taken from babies that were symptomatic and/or outside the ‘newborn’ period. There were significant concerns regarding applicability of the research to the UK screening population in seven studies as the incidence of TYR1 was higher than expected in the UK population and/or screened dried blood spot samples were collected before five days or after eight days of life [14, 17–20, 22, 24].

In the index test domain, five studies were judged to have a high risk of bias as the results of the reference test were known when interpreting the index test in all case–control studies and the SUAC threshold was not pre-specified [14, 21–24]. Applicability concerns were low for all ten studies.

In the reference test domain, two studies were judged to be at high risk of bias as case-controls received a screening test (second-tier test measuring SUAC directly or indirectly in babies with elevated tyrosine levels) as a reference standard [23, 24]. The remainder of the studies had an unclear risk of bias as they did not report the method of diagnosis, or did not report sufficient information to allow a judgement to be made [12, 14, 17–22]. Applicability concerns were high in seven studies as babies that screened negative in studies reporting screening experiences or were used as controls did not receive an appropriate reference standard, i.e. diagnostic testing or clinical follow-up for at least two years [12, 17–20, 23, 24].

In the flow and timing domain, all ten studies were considered to be at high risk of bias. The reasons for this were that the reference standards used to confirm TYR1 status for screen-positives and screen negatives (or cases and controls) were not the same, follow-up of those people who screened-negative was not defined or not conducted, and losses to follow-up were not reported [12, 14, 17–24].

### Characteristics of included studies

Included studies are summarised in table 1 and Additional file 1: supplement 6. There were ten studies. Five studies reported experiences of newborn screening programmes [12, 17–20]. Data were given for screening periods ranging from 16 weeks [19] to four years one month [20]; the number of analysed screening samples ranged from 61,344, which included two cases [19], to 518,687, which included three cases [20]. Five papers reported on case–control studies [14, 21–24] conducted over periods between one [21] and five [22] months; the number of screening samples ranged from ~1000, which included six cases [21], to 13,532, which included 11 cases [24].

**Table 1:** Accuracy of newborn screening tests using MS/MS measurement of SUAC

Study	No. screened	SUAC cut-off	2×2 table				Sensitivity% (95% CI)	Specificity% (95% CI)	PPV% (95% CI)	NPV% (95% CI)
			TP	TN	FP	FN				
Studies reporting screening experiences										
La Marca [17]	136,075 [Overlap of 13,000 with [22]]	2 μmol/l	2	NA	0	NA	NA	NA	100 (19.8-100)	NA
Lund [18]	140,565	2.1 U	1	NA	0	NA	NA	NA	100 (5.5-100)	NA
Morrissey [12]	~500,000	3 μmol/l	2	NA	1	NA	NA	NA	66.7 (12.5-98.2)	NA
Sander [19]	61,344	≥10 μmol/l	2	NA	0	NA	NA	NA	100 (19.8-100)	NA
Zytkovicz [20]	518,687: 515,592 newborns, 3095 over 1 month old	>4 μM (recently reduced to 3.3 μM) positive; SUAC 1.0-3.3 μM intermediate	*3*	NA	0	NA	NA	NA	100 (31.0-100)	NA
Case–control studies										
Allard [14]	4,002: 3 cases, 3,199 controls	2 μmol/l	3	3199	0	0	100 (31.0-100)	100 (99.85-100)	N/A	N/A
Dhillon [21]	>1,026: 6 cases, >1,000 controls	3 μmol/l	6	>1,000	0	0	100 (51.7-100)	N/A	N/A	N/A
La Marca [22]	13,006: 6 cases, 13,000 controls [Overlap with la Marca [17]]	2.4 μmol/l	6	13,000	0	0	100 (51.7-100)	100 (99.96-100)	N/A	N/A
Metz [23]	4686: 3 cases, 4683 controls	1.29 μmol/l	3	4683	0	0	100 (30.9-100)	100 (99.90 – 100)	N/A	N/A
Turgeon [24]	13,532: 11 cases, 13,521 controls.	>5 μmol/l	11	13,521	0	0	100 (67.9-100)	100 (99.96-100)	N/A	N/A

### Description of screening and diagnostic tests

Details of the MS/MS screening methodology and diagnostic confirmation used in the included studies are provided in Additional file 1: supplement 6. In brief, two studies used commercially available MS/MS assays [18, 23] while all others used non-kit methods with derivatisation of SUAC to its hydrazine [12, 14, 17, 19–22, 24]. MS/MS analysis of SUAC-hydrazone [12, 14, 19, 20, 24] or SUAC-hydrazone butyl ester [17, 21–23] was performed; the MS/MS methodology used was not reported by Lund et al. [18]. The SUAC cut-off values used in the 10 studies ranged from 1.29 μmol/l [23] to 10 μmol/l [19]. No two studies used the same cut-off value.

A range of approaches were reported for defining the reference standard. For individuals who screened positive or were used as cases these were: clinical diagnosis [14], “pre-natal testing” [12], DNA analysis [17, 18], analysis of SUAC in urine [12, 18, 19], analysis of plasma amino acids [12, 18], “diagnostically confirmed in accordance with institutional guidelines” [23], and on the basis of “symptoms consistent with TYR1” [20]. The method of diagnosis was not reported by Dhillon and colleagues [21] and la Marca and colleagues [22]. Four studies used more than one diagnostic approach [12, 18–20]. For individuals who screened negative, no clinical follow-up or other reference standard was reported for all five experience reports [12, 17–20]. In the five case–control studies, two conducted second-tier testing (SUAC or 5-aminolevulinic acid dehydratase [ALAD]) in DBS with elevated tyrosine levels to identify healthy controls [23, 24], while the reference standard to confirm absence of TYR1 was unclear in the other three studies [14, 21, 22].

#### Accuracy of screening tests

The methods and thresholds used for screening, and diagnostic tests varied between studies. Results were considered positive when they exceeded the threshold as set in the individual study. Table 1 shows the test accuracy data on sensitivity, specificity, positive predictive value (PPV) and negative predictive value (NPV).

### Sensitivity and specificity

It was not possible to calculate sensitivity and specificity for the studies reporting screening experiences due to a lack of follow-up of people who had screened negative. For the case–control studies, sensitivity was estimated to be 100% in each of the five studies, which included 29 cases in total [14, 17, 21, 23, 24]. Specificity was estimated to be 100% in four studies [14, 22–24]. This included 34,403 unaffected babies in total, 18,204 of which had an inadequate reference standard. Specificity could not be calculated for the study by Dhillon et al. [21] as they did not give a precise figure for the number of controls.

### Positive and negative predictive value

In the studies reporting screening experiences, the PPV was 100% in four studies, with in total eight true positive cases and no false positive cases out of 856,671 people screened [17–20], and 66.7% in one study, with two true positive cases and one false positive case out of ~500,000 babies screened [12]. There were very wide confidence intervals due to the small number of cases. NPV could not be calculated due to a lack of follow-up of people who had screened negative. PPV and NPV could not be calculated from the case–control studies as these values are dependent on the prevalence of the disease in the population that is being tested.

## Discussion

We examined the test accuracy of SUAC measurement in DBS using MS/MS to screen for TYR1 in newborns. Ten studies were identified which reported test accuracy data; five studies reporting screening experiences and five case–control studies. PPV in the studies reporting screening experiences ranged from 67% (two true positive cases and one false positive case out of ~500,000 babies screened) to 100% (eight true positive cases and no false positive cases out of 856,671 people screened). We were unable to calculate sensitivity, specificity, or negative predictive value in these studies due to a lack of follow-up of babies who screened negative. Case–control studies reported clear discrimination between SUAC levels of newborns with and without TYR1.

No consistent test accuracy metric was available. Papers reporting screening experiences suggested that using SUAC to screen for TYR1 resulted in no false negative results, and reported test sensitivity and specificity of up to 100%. However, these conclusions were based on a lack of awareness of false negative results rather than following up babies who had screened negative. Without proper follow-up of the population who have been tested, for an appropriate amount of time, it is not possible to know if the absence of awareness of false negatives reflects an actual absence of false negative results.

While case–control studies showed no overlap in SUAC levels between newborns with and without TYR1, the cut-offs used varied between studies and were specified retrospectively, and the assessors were not blinded to the disease status, which can result in overestimation of test accuracy. The included case–control studies were also at high or unclear risk of differential verification bias as TYR1 cases and healthy controls received different reference standards, the reference standards used were not reported in sufficient detail to assess if their accuracy was comparable, or they were not reported at all. The use of multiple reference standards across participants of a single study might have resulted in an overestimation of accuracies [25]. In addition, studies evaluating diagnostic tests in a diseased population and a separate healthy control group can overestimate the diagnostic performance compared with studies that use the index test in a clinical population covering the full range of patients without knowing their disease status [25].

Our understanding of the appropriateness of screening for TYR1 using SUAC is limited by heterogeneity in study design, the methods used for SUAC determination on DBS, and the SUAC cut-off values. For example, the SUAC cut-offs used in the screening test to identify possible cases of TYR1 ranged from 1.29 μmol/l [23] to 10 μmol/l [19]. Proficiency testing results for SUAC in dried blood spots have shown large differences among screening laboratories in SUAC recovery reflecting analytic biases, which might explain the wide variation in cut-off values of the studies in our review [26]. Differences in recovery could be explained by the method used (kit TMS vs. non-kit TMS; butyl ester derivatisation vs. non-derivatisation), DBS extraction strategy (freshly punched DBS, residual DBS or co-extraction of AA, AC, and SUAC, respectively), internal standard used (^13^C-SUAC, 5,7-dioxooctanoic acid, or TMS kit internal standard), or the calibration strategy used (DBS calibrators, TMS internal standard/other liquid standard or kit internal standard only, respectively). Laboratories that measure low quantitative SUAC results usually used lower cut-off values to avoid misclassifications [26]. This highlights an important issue in how screening tests are evaluated. In this paper, we examine test accuracy, meaning the association between results from the test under investigation with the presence or absence of the target disease. However, the term ‘accuracy’ has multiple meanings. Within method validation (the process used to confirm that tests are suitable for their intended purpose), ‘analytical’ accuracy refers to the degree to which test results and the true value of the measured quantity agree and how reproducible and reliable the test is [27]. The analytical performance of the used SUAC assays has been described in some of the included studies. The recovery of SUAC was assessed in five studies by assaying DBS specimens enriched with predetermined (low to high) SUAC concentrations and was reported to be 51% [23], 72-80% [19], 75-78% [14], 75-86% [21], and 97-100% [22] of the expected value, respectively. The quantification limit (the lowest amount of SUAC in a sample which can be reliably quantified) was reported in four studies and was 0.4 μmol/l [22], 0.5 μmol/l [19, 23] and 1 μmol/l [14]. The calibration was reported to be linear up to 50 μmol/l [14], 100 μmol/l [19, 22, 24], 240 μmol/l [21], and 250 μmol/l [23], respectively. Precision (the ability to consistently reproduce a result when sub-samples are taken from the same specimen) results were presented in seven studies with inter-assay coefficients of variation (CV) at different SUAC concentrations of 10.0-12.2% [14], 7.1-8.5% [21], 3.50-4.49% [22], 5.8-13% [19], 15.8-16.7% [24], 17.29-19.00% [23] and 30% in a pooled sample assay [20]. Taken together, the analytical performance of the screening tests used in the included studies was in agreement with previously reported proficiency testing outcomes [26, 28, 29], showing large between-laboratory differences in SUAC recoveries (mostly incomplete recoveries) depending on the method used and reproducible within-laboratory recoveries. There is need to harmonise quantitative results among laboratories. Despite differences among methods in SUAC recoveries (analytical bias), each method seems to have an acceptable precision and might therefore still be able (when using a cutoff value appropriate for the selected method) to reliably sort asymptomatic newborns into probable TYR1 cases and non-cases. De Jesus et al. [29] and Adam et al. [26] stress in their papers that bias in quantitative results can be tolerated if the screening test reliably sorts people into those who (probably) do have the disease of interest and those who (probably) don’t. Any differences in the test accuracy between studies might be due to the timing of the test, the SUAC assay used, the cut-off used for classifying the disease status, use of repeat testing in samples with borderline SUAC levels, or variation in normal SUAC values in the tested newborn population.

Our review has a number of limitations. First, we were unable to synthesise our findings numerically due to incomplete 2x2 tables for reporting screening experiences, and heterogeneity in study design, the MS/MS method used, and the SUAC cut-off values. Second, we restricted our search to English language papers; non-English-language papers may be available and add further information. Third, we tailored the applicability questions for the QUADAS-2 in relation to the newborn screening in the UK. For example, in the UK newborn screening takes place five to eight days after birth, so studies in which samples were taken before or after this were rated as having high concerns regarding applicability. None of the studies we identified were conducted in the UK, and the usual time at which screening takes place varies by country; in many European countries newborn screening is conducted three days after birth. Therefore, the criteria for a high applicability concern might be different outside the UK.

While results from case–control studies are promising they are not definitive, as we know that case–control designs tend to overestimate test accuracy [25]. A research project using MS/MS measurement of SUAC from DBS with follow-up of screen-negatives for at least two years would considerably strengthen the test accuracy data. This could be achieved by following up one of the existing cohorts described in this review by searching hospital/primary care databases for cases of TYR1 that were identified symptomatically. While this approach would not provide a definitive answer, it would enable a measure of false-negative cases that is currently missing from the literature.

## Conclusions

MS/MS measurement of SUAC from DBS looks like a promising screening test for TYR1 but test accuracy from proof-of-concept studies should be confirmed in screening studies that include appropriate follow-up of screen-negatives.

## Additional file

Additional file 1: Supplement 1 Search strategy for Ovid Medline (26th January 2016). Supplement 2 QUADAS-2 checklist. Supplement 3 Modified QUADAS-2 and guidance notes for tyrosinaemia type 1 screening. Supplement 4 Excluded studies (n=66). Supplement 5 Risk of bias and applicability concerns summary: review authors' judgements about each domain for each included study. Supplement 6 Study characteristics and MS/MS screening methodology for Tyrosinemia type 1. (DOCX 78 kb)

## Abbreviations

ALAD: 5-aminolevulinic acid dehydratase
CV: Coefficient of variation
DBS: Dried blood spot
MS/MS: Tandem mass spectrometry
NPV: Negative predictive value
PPV: Positive predictive value
QUADAS-2: Quality assessment tool for diagnostic accuracy studies 2
SUAC: Succinylacetone
TYR1: Tyrosinemia type 1
