# Supplementary material for: Newborn screening for Tyrosinemia type 1 using succinylacetone – a systematic review of test accuracy
Source: Orphanet J Rare Dis. 2017 Mar 9;12:48. doi: 10.1186/s13023-017-0599-z (PMC5343414; doi:10.1186/s13023-017-0599-z)
Supplement: Additional file 1: — Supplement 1 Search strategy for Ovid Medline (26th January 2016). Supplement 2 QUADAS-2 checklist. Supplement 3 Modified QUADAS-2 and guidance notes for tyrosinaemia type 1 screening. Supplement 4 Excluded studies (n=66). Supplement 5 Risk of bias and applicability concerns summary: review authors' judgements about each domain for each included study. Supplement 6 Study characteristics and MS/MS screening methodology for Tyrosinemia type 1. (DOCX 78 kb) [file 13023_2017_599_MOESM1_ESM.docx]

| **Supplement 1**. Search strategy for Ovid Medline (26th January 2016). | | |
| --- | --- | --- |
| # | Search | Results |
| 1 | exp Tyrosinemias/ | 309 |
| 2 | (tyrosinemia* or tyrosinaemia*).mp. [mp=title, abstract, original title, name of substance word, subject heading word, keyword heading word, protocol supplementary concept word, rare disease supplementary concept word, unique identifier] | 989 |
| 3 | (tyr1 or tyr-1).mp. [mp=title, abstract, original title, name of substance word, subject heading word, keyword heading word, protocol supplementary concept word, rare disease supplementary concept word, unique identifier] | 961 |
| 4 | (tyri or tyr-i).mp. [mp=title, abstract, original title, name of substance word, subject heading word, keyword heading word, protocol supplementary concept word, rare disease supplementary concept word, unique identifier] | 28 |
| 5 | (((fumarylacetoacetate adj hydrolase) or fumarylacetoacetase or fah) adj2 deficien*).mp. [mp=title, abstract, original title, name of substance word, subject heading word, keyword heading word, protocol supplementary concept word, rare disease supplementary concept word, unique identifier] | 122 |
| 6 | metabolism, inborn errors/ or exp amino acid metabolism, inborn errors/ or exp amino acid transport disorders, inborn/ | 32709 |
| 7 | inborn metabolic disorder*.mp. | 70 |
| 8 | inborn metabolic error*.mp. | 71 |
| 9 | 1 or 2 or 3 or 4 or 5 or 6 or 7 or 8 | 34016 |
| 10 | suac.mp. | 23 |
| 11 | exp Heptanoates/ or succinylacetone.mp. | 425 |
| 12 | succinylacetoacetate.mp. | 13 |
| 13 | 51568 18 4 succinylacetone.rn. | 218 |
| 14 | 4,6-dioxoheptanoate.mp. | 4 |
| 15 | exp Heptanoic Acids/ or 4,6-Dioxoheptanoic acid.mp. | 6027 |
| 16 | 10 or 11 or 12 or 13 or 14 or 15 | 6145 |
| 17 | tandem mass spectrometry.mp. or exp Mass Spectrometry/ or exp Tandem Mass Spectrometry/ | 194658 |
| 18 | mass spectro*.mp. | 216542 |
| 19 | tms.mp. | 7402 |
| 20 | 17 or 18 or 19 | 243854 |
| 21 | exp Dried Blood Spot Testing/ or (Blood spot* or dry blood spot* or dried blood spot*).mp. | 3297 |
| 22 | dbs.mp. | 5020 |
| 23 | 21 or 22 | 7449 |
| 24 | neonatal screening.mp. or exp Neonatal Screening/ | 8921 |
| 25 | neonat* screening.mp. | 8930 |
| 26 | newborn screening.mp. | 3462 |
| 27 | ((newborn* or neonat*) adj5 screen*).mp. [mp=title, abstract, original title, name of substance word, subject heading word, keyword heading word, protocol supplementary concept word, rare disease supplementary concept word, unique identifier] | 12346 |
| 28 | 24 or 25 or 26 or 27 | 12346 |
| 29 | 20 and 28 | 821 |
| 30 | 16 or 29 or 23 | 14082 |
| 31 | 9 and 30 | 729 |

**Supplement 2.** QUADAS-2 checklist

**QUADAS-2 (adjusted)**

*First author surname and year of publication:*

*Name of first reviewer: Name of second reviewer:*

**Phase 1: State the review question:**

2) What is the test accuracy (sensitivity, specificity, and predictive values applicable to UK prevalence) of SUAC measurement in dried blood spots using TMS for TYR1 screening?

| *Patients (setting, intended use of index test, presentation, prior testing):* |
| --- |
| *Index test(s):* |
| *Reference standard and target condition:* |

**Phase 2: Draw a flow diagram for the primary study**

|  |
| --- |

**Phase 3: Risk of bias and applicability judgments**

*QUADAS-2 is structured so that 4 key domains are each rated in terms of the risk of bias and the concern regarding applicability to the research question (as defined above). Each key domain has a set of signalling questions to help reach the judgments regarding bias and applicability.*

| **DOMAIN 1: PATIENT SELECTION**   1. **Risk of Bias** | |
| --- | --- |
| Describe methods of patient selection: | |
| + Was a consecutive or random sample of patients enrolled? | Yes/No/Unclear |
| + Was a case-control design avoided? | Yes/No/Unclear |
| + Did the study avoid inappropriate exclusions? | Yes/No/Unclear |
| + Were screening samples taken in asymptomatic babies? | Yes/No/Unclear |
| **Could the selection of patients have introduced bias?** | **RISK: LOW/HIGH/UNCLEAR** |
| 1. **Concerns regarding applicability** | |
| Describe included patients (prior testing, presentation, intended use of index test and setting)**:** | |
| **Is there concern that the included patients do not match** **the review question?** | **CONCERN: LOW/HIGH/UNCLEAR** |

| **DOMAIN 2: INDEX TEST(S)**  **If more than one index test was used, please complete for each test.**   1. **Risk of Bias** | |
| --- | --- |
| Describe the index test and how it was conducted and interpreted: | |
| + Were the index test results interpreted without knowledge   of the results of the reference standard? | Yes/No/Unclear |
| + If a threshold was used, was it pre-specified? | Yes/No/Unclear |
| **Could the conduct or interpretation of the index test have introduced bias?** | **RISK: LOW/HIGH/UNCLEAR** |
| 1. **Concerns regarding applicability** | |
| **Is there concern that the index test, its conduct, or interpretation differ from the review question?** | **CONCERN: LOW/HIGH/UNCLEAR** |

| **DOMAIN 3: REFERENCE STANDARD**   1. **Risk of Bias** | |
| --- | --- |
| Describe the reference standard and how it was conducted and interpreted: | |
| + Is the reference standard likely to correctly classify the   target condition? | Yes/No/Unclear |
| + Were the reference standard results interpreted without   knowledge of the results of the index test? | Yes/No/Unclear |
| **Could the reference standard, its conduct, or its interpretation have introduced bias?** | **RISK: LOW/HIGH/UNCLEAR** |
| 1. **Concerns regarding applicability** | |
| **Is there concern that the target condition as defined by the reference standard does not match the review question?** | **CONCERN: LOW/HIGH/UNCLEAR** |

| **DOMAIN 4: FLOW AND TIMING**   1. **Risk of Bias** | |
| --- | --- |
| Describe any patients who did not receive the index test(s) and/or reference standard or who were excluded from the 2x2 table (refer to flow diagram): | |
| Describe the time interval and any intervention between index tests(s) and reference standard: | |
| + Was there an appropriate interval between index test(s) and reference standard? | Yes/No/Unclear |
| + Did all patients receive a reference standard? | Yes/No/Unclear |
| + Did all patients receive the same reference standard? | Yes/No/Unclear |
| + Were all patients included in the analysis? | Yes/No/Unclear |
| **Could the patient flow have introduced bias?** | **RISK: LOW/HIGH/UNCLEAR** |

**Supplement 3.** Modified QUADAS-2 and guidance notes for tyrosinaemia type 1 screening

**Domain 1: Patient selection**

SUAC levels in symptomatic older children might be different to levels in asymptomatic patients in the newborn period . Therefore choice of patient population in terms of disease status is an important factor for consideration for bias and applicability.

A. Risk of bias

Guidance:

*Was a consecutive or random sample of patients enrolled?*

This question should only be answered with ‘yes’ if the study clearly states that newborn babies (rather than samples) were recruited consecutively or randomly.

*Was a case-control design avoided?*

We would at least expect a prospective cohort design. Therefore, if the study is a case-control study this question should be answered with ‘No’.

*Did the study avoid inappropriate exclusions?*

If the study excludes >10% of participants with or without specifying reasons, the exclusions should be considered as inappropriate. This cut-off has been determined pragmatically. If studies reporting experiences of implementing screening have a reported screening uptake of at least 95% this question can be answered with ‘yes’.

*Were screening samples taken in asymptomatic babies?*

This question can be answered with ‘yes’ if all samples were taken in babies without clinical symptoms of TYR I. The question should be answered with ‘no’ if symptomatic children were tested and the risk of bias classed as ‘high’.

B. Concerns regarding applicability

Guidance:

The timing of sample collection might influence test performance. As the research question aims to address screening of newborns within NHS schedules of 5-8 days, sampling outside this window, e.g. at 3 days (many European countries including Ireland, Italy, Denmark) might not produce outcomes applicable to the NHS context. Likewise, a cohort study in a country with significantly different prevalence of cases will affect applicability.

Applicability concerns should be regarded ‘high’ if >10% of blood spot specimens were collected in babies >10 days or < 5 days.

Applicability concerns should be also considered ‘high’ if TYR1 prevalence in cohort studies differs significantly from that expected in UK newborns (~1:100,000) , i.e. in countries: Québec province of Canada, parts of Finland and Norway, Pakistan, Middle East and North Africa .

**Domain 2: Index test**

The main sources of bias introduced by conducting and interpreting the index test are blinding and defining the threshold. If the reference standard is carried out before the index test (e.g. in case control studies) it is important to blind personnel to the results of the reference standard.

The QUADAS-2 tool requires a threshold to be pre-specified in the methods in order to avoid adjustment of the threshold according to the test outcome.

1. Risk of bias

*Were the index test results interpreted without knowledge of the results of the reference standard?*

In case-control design studies blinding needs to be specifically mentioned for this question to be answered with ‘yes’.

*Was a threshold explicitly pre-specified?*

For this question to be answered with ‘yes’ the study needs to mention what kind of threshold was to be used (e.g. SUAC ≥2.0 µmol/l) and clearly state that it was specified before the start of the study. If the study reports adjustment to the threshold and reports results according to adjusted thresholds this question should be answered with ‘no’.

B. Concerns about applicability

If the study uses different screening tests to TMS measurement of SUAC as primary marker (i.e. SUAC as 2^nd^-tier test) concerns regarding the applicability of the study should be classed as ‘high’.

**Domain 3: Reference standard**

The most useful test for diagnosis of TYR1 is measurement of SUAC in plasma, dried blood spot or urine by a specific assay, which is highly specific and sensitive. SUAC may not be detected in routine organic analyses in urine, particularly if the concentration of SUAC is very low or the urine is very dilute. Molecular genetic testing for common FAH pathogenic variants or detection of decreased activity of the FAH enzyme in liver tissue or cultured fibroblasts might also be used for diagnosis (<http://www.ncbi.nlm.nih.gov/books/NBK1515)> but is generally used to support SUAC testing and is unlikely used as the initial test of investigation because it is still expensive and deletions are not always.

Plasma amino acids can only be seen as supportive of a diagnosis as they are very nonspecific.

Follow-up as a reference standard should be for at least two years to confirm absence of TYR1 .

Furthermore, blinding is an issue in studies reporting experiences as only samples with high SUAC samples will be referred to further testing. If the index test is carried out before the reference standard, blinding to the results of the index tests is important.

1. Risk of bias

*Is the reference standard likely to correctly classify the target condition?*

For studies that used SUAC levels in urine, molecular genetic testing or at least 2 year follow-up as reference standard on their own or in combination this question should be answered with ‘yes’. If studies only used plasma amino acids (Tyr) levels as a reference standard the question should be answered with ‘no’. If studies using SUAC in urine as the reference standard do not define the SUAC threshold used the question should be answered with ‘unclear’.

*Were the reference standard results interpreted without knowledge of the results of the index test?*

This question should be answered with ‘no’ if the study reports an experience report. In case-control studies blinding of the index test results is not an issue and can therefore be classed as ‘yes’. In cohort studies blinding should be specifically mentioned for the question to be answered with ‘yes’.

B. Concerns about applicability

The concern of applicability of the reference standard will be ‘low’ if SUAC levels in urine, molecular genetic testing or at least 2 year follow-up were the predefined reference standard in the studies assuming that they all identify TYR1 and no other type of tyrosinaemia.

**Domain 4: Flow and Timing**

Innate residual FAH activity as well as protein (especially phenylalanine) intake have been shown to influence production of SUAC in TYR1 cases . SUAC usually accumulates over time in effected cases and late testing is therefore associated with better test performance. On the other hand there are also reports that SUAC concentrations in asymptomatic newborns were higher than in symptomatic older infants when hepatic and renal dysfunction has set in . Since TYR1 is a progressive condition, the time interval between the index test and reference standard is of importance. Partial and differential verification bias are a concern in studies where screen-negative children are not referred for further testing and receive no reference standard (experience reports) or are followed up for a certain time period only (cohort studies) as these studies have limited data on false negatives.

Children with negative index test result should be followed up for at least two years to confirm absence of TYR1 and losses to follow-up should be reported.

1. Risk of bias

*Was there an appropriate interval between index test(s) and reference standard?*

This question can be answered with ‘yes’ if the reference standard is SUAC in urine and the time interval is <10 days. This number has been chosen arbitrarily on the basis that small molecules have been shown to increase from undetectable to detectable levels between birth and two to four days of life. It is very likely that bias will exist if the index test and reference standard (both measuring SUAC) are not undertaken at the same time. However, no evidence was identified to base this cut-off on. The question should be answered with ‘no’ if treatment commenced between index test and references standard that decreases the level of SUAC and bias should be regarded as ‘high’. The question can be answered with ‘yes’ if molecular testing or follow-up were used as the reference standard.

*Did all patients receive a reference standard?*

This question can be answered with ‘yes’ if all participants are recruited on the basis of the above mentioned reference standard results (case-control studies). In prospective cohort studies, this question can be answered with ‘yes’ if all those who screen positive have received one of the above mentioned reference standards and those who screened negative were followed up for at least two years and losses to follow-up are reported and are <10%.

The question should be answered with ‘unclear’ if the study provides no information on how healthy controls were identified in case-control studies and risk of bias should be classed as ‘high’.

Risk of bias should be considered as ‘high’ in case-control studies which included healthy controls identified on the basis of newborn screening results (e.g. two tier screening of Tyr levels followed by SUAC in dried blood spots) and/or follow-up for less than two years; or in prospective cohort studies which followed up screen negatives for less than two years.

Risk of bias should be considered as ‘high’ in studies that did not specify and describe (including threshold for SUAC levels in urine) the reference standard.

*Did all patients receive the same reference standard?*

This question should be answered with ‘no’ if patients received different reference standards or if positive cases received a different reference standard to negative subjects. This question should also be answered with ‘no’ if a list of reference standards is given but no report is made of which patients received which reference standard(s). If this question is answered with ‘no’, the risk of bias should be regarded as ‘high’ as the different reference standards cannot be classed as equivalent.

*Were all patients included in the analysis?*

If inconclusive or intermediate results are not considered in the analysis the question should be answered with ‘no’. If patients lost to follow-up were not included in the analysis or >50% of patients were lost to follow (even if considered in the analysis) the question should be answered with ‘no’. (The actual proportion of patients lost to follow-up needs to be recorded for each study). If studies report a clinical experience and base test accuracy estimates on interim results and not all patients were followed up the question should be answered with ‘no’. In all three cases the risk of bias should be classed as ‘high’.

| **Supplement 4.** Excluded studies (n=66) | |
| --- | --- |
| Reference | Reason for exclusion |
| Abdel-Hamid M. Tisocki K. Sharaf L. Ramadan D. 2007. Development, validation and application of tandem mass spectrometry for screening of inborn metabolic disorders in Kuwaiti infants. *Medical Principles & Practice* **16**: 215-221. | Not SUAC as primary marker |
| Arn PH. 2007. Newborn screening: current status. *Health Affairs* **26**: 559-566. | No test accuracy data |
| Bennett MJ. 2013. "The expansion of biomarker measurement for metabolic disease diagnosis." *Annals of Clinical Biochemistry* **50**: 386-387. | No test accuracy data for TYR1 screening |
| Bodamer, O. A., et al. 2007. Expanded newborn screening in Europe 2007. *Journal of Inherited Metabolic Disease* **30**: 439-444. | No test accuracy data |
| Bodamer OA. Muhl A. 2005. Analysis of acylcarnitine ester for neonatal screening of inborn errors of metabolism using tandem mass-spectrometry. *Monatshefte Fur Chemie* **1368**: 1293-1297. | Not TYR1 screening using SUAC as primary marker |
| Bulbul S. 2014. Novel approach for Newborn Errors in Metabolism Screening (NEMS) by NMR: Clinical NEMS-by-NMR Study in Turkey. *Clinical Biochemistry* **47**: 700-701. | NMR analysis of urine samples |
| Chace DH, Hannon WH. 2010. Impact of second-tier testing on the effectiveness of newborn screening. *Clinical Chemistry* **56**: 1653-1655. | Editorial |
| Champion MP. 2010. An approach to the diagnosis of inherited metabolic disease. *Archives of Disease in Childhood Education & Practice* **95**: 40-46. | No test accuracy data |
| Couce ML. Castiñeiras DE. Bóveda MD. Baña A. Cocho JA. Iglesias AJ. Colón C. Alonso-Fernández JR. Fraga JM. 2011. Evaluation and long-term follow-up of infants with inborn errors of metabolism identified in an expanded screening programme. *Molecular Genetics & Metabolism* **104**: 470-475. | Tyrosine as primary screening marker |
| Denes J. Szabó E. Robinette SL. Szatmári I. Szőnyi L. Kreuder JG. Rauterberg EW. Takáts Z. 2012. Metabonomics of newborn screening dried blood spot samples: a novel approach in the screening and diagnostics of inborn errors of metabolism. *Analytical Chemistry* **84**: 10113-10120. | No test accuracy data for TYR1 screening |
| Eddy M. Gottesman GS. 2009. Newborn metabolic screening and related pitfalls. *Missouri Medicine* **106**: 234-240. | No test accuracy data for TYR1 screening |
| Fernhoff P. M. 2009. Newborn screening for genetic disorders. *Pediatric Clinics of North America* **56**: 505-513. | No test accuracy data for TYR1 screening |
| Feuchtbaum L. Dowray S, Lorey F. 2010. The context and approach for the California newborn screening short- and long-term follow-up data system: Preliminary findings. *Genetics in Medicine* **12**: S242-S250. | No test accuracy data for TYR1 screening |
| Fingerhut R. Olgemoller B. 2009. Newborn screening for inborn errors of metabolism and endocrinopathies: an update. *Analytical & Bioanalytical Chemistry* **393**: 1481-1497. | No test accuracy data for TYR1 screening |
| Frazier DM. Millington DS. McCandless SE. Koeberl DD. Weavil SD. Chaing SH. Muenzer J.2006. The tandem mass spectrometry newborn screening experience in North Carolina: 1997-2005. *Journal of Inherited Metabolic Disease* **29**: 76-85. | Tyrosine as marker analyte |
| Gu X. Wang Z. Ye J. Han L. Qiu W. 2008. Newborn screening in China: phenylketonuria, congenital hypothyroidism and expanded screening. *Annals of the Academy of Medicine*, Singapore **37**: 107-104. | No test accuracy data for TYR1 screening |
| Harms E. Olgemoeller B. 2011. Neonatal Screening for Metabolic and Endocrine Disorders. *Deutsches Arzteblatt International* **108**: 11-21. | No test accuracy data for TYR1 screening |
| Hsu KP. Hsieh SH. Hsieh SL. Cheng PH. Weng YC. Wu JH. Lai F. 2010. A newborn screening system based on service-oriented architecture embedded support vector machine. *Journal of Medical Systems* **34**: 899-907. | No test accuracy data for TYR1 screening |
| Huang HP. Chu KL. Chien YH. Wei ML. Wu ST. Wang SF. Hwu WL. 2006. Tandem mass neonatal screening in Taiwan - Report from one center. *Journal of the Formosan Medical Association* **105**: 882-886. | Not SUAC as primary marker |
| James PM. Levy HL. 2006. The clinical aspects of newborn screening: Importance of newborn screening follow-up. *Mental Retardation and Developmental Disabilities Research Reviews* **12**: 246-254. | No test accuracy data for TYR1 screening |
| Joseph R. 2005. Screening newborns for Inborn Errors of Metabolism - A standard of care. *Perinatology* **7**: 243-245. | No test accuracy data for TYR1 screening |
| Joshi SN. Venugopalan P. 2007. Clinical characteristics of neonates with inborn errors of metabolism detected by Tandem MS analysis in Oman. *Brain & Development* **29**: 543-546. | Not SUAC as primary marker |
| Kasper DC. Ratschmann R. Metz TF. Mechtler TP. Möslinger D. Konstantopoulou V. Item CB. Pollak A. Herkner KR. 2010. The national Austrian newborn screening program - eight years’ experience with mass spectrometry. past, present, and future goals. *Wiener Klinische Wochenschrift* **122**: 607-613. | Tyrosine as primary marker and ALAD activity as second-tier test |
| Khneisser, I., et al. 2015. Cost-benefit analysis: Newborn screening for inborn errors of metabolism in Lebanon. *Journal of Medical Screening* **22**: 182-186. | No test accuracy data for TYR1 screening |
| Khneisser I. Adib S. Assaad S. Megarbane A. Karam 4. 2008. International cooperation in the expansion of a newborn screening programme in Lebanon: a possible model for other programmes. *Journal of Inherited Metabolic Disease* **31**: S441-446. | Tyrosine as marker analyte |
| la Marca, G. 2014. Mass spectrometry in clinical chemistry: the case of newborn screening. *Journal of Pharmaceutical and Biomedical Analysis* **101**: 174-182. | No test accuracy data for TYR1 screening |
| la Marca G. Malvagia S. Pasquini E. Cavicchi C. Morrone A. Ciani F. Funghini S. Villanelli F. Zammarchi E. Guerrini R. 2011. Newborn Screening for Tyrosinemia Type I: Further Evidence that Succinylacetone Determination on Blood Spot Is Essential. *Jimd Reports* **1**: 107-109. | Duplicate |
| la Marca G. Malvagia S. Casetta B. Pasquini E. Donati MA. Zammarchi E. 2008. Progress in expanded newborn screening for metabolic conditions by LC-MS/MS in Tuscany: update on methods to reduce false tests. *Journal of Inherited Metabolic Disease* **31**: S395-404. | No separate test accuracy data for TYR1 screening using SUAC as primary marker |
| Lim JS. Tan ES. John CM. Poh S. Yeo SJ. Ang JS. Adakalaisamy P. Rozalli RA. Hart C. Tan ET. Ranieri E. Rajadurai VS. Cleary MA. Goh DL. 2014. Inborn Error of Metabolism (IEM) screening in Singapore by electrospray ionization-tandem mass spectrometry (ESI/MS/MS): An 8 year journey from pilot to current program. *Molecular Genetics & Metabolism* **113**: 53-61. | SUAC as 2^nd^-tier test |
| Lindner M. Gramer G. Haege G. Fang-Hoffmann J. Schwab KO. Tacke U. Trefz FK. Mengel E. Wendel U. Leichsenring M. Burgard P. Hoffmann GF. 2011. Efficacy and outcome of expanded newborn screening for metabolic diseases--report of 10 years from South-West Germany. *Orphanet Journal Of Rare Diseases* **6**: 44. | Tyrosine as primary marker and spectrophotometric microassay for ALAD as 2^nd^-tier test |
| Lodh M. Kerketta A. 2013. Inborn errors of metabolism in a tertiary care hospital of eastern India. *Indian Pediatrics* **50**: 1155-1156. | No test accuracy data for TYR1 screening |
| Loukas YL. Soumelas GS. Dotsikas Y. Georgiou V. Molou E. Thodi G. Boutsini M. Biti S. Papadopoulos K. 2010. Expanded newborn screening in Greece: 30 months of experience. *Journal of Inherited Metabolic Disease* **33**: S341-348. | Tyrosine as primary marker and SUAC as 2^nd^-tier test, no test accuracy data |
| Lukacs Z. Santer R. 2006. Evaluation of electrospray-tandem mass spectrometry for the detection of phenylketonuria and other rare disorders. *Molecular Nutrition & Food Research* **50**: 443-450. | No test accuracy data for TYR1 screening |
| Lund AM. Hougaard DM. Simonsen H. Andresen BS. Christensen M. Dunø M. Skogstrand K. Olsen RK. Jensen UG. Cohen A. Larsen N. Saugmann-Jensen P. Gregersen N. Brandt NJ. Christensen E. Skovby F. Nørgaard-Pedersen B. 2012. Biochemical screening of 504,049 newborns in Denmark, the Faroe Islands and Greenland - Experience and development of a routine program for expanded newborn screening. *Molecular Genetics and Metabolism* **107**: 281-293. | Duplicate |
| Luz Couce M. Castiñeiras DE. Bóveda MD. Baña A. Cocho JA. Iglesias AJ. Colón C. Alonso-Fernández JR. Fraga JM. 2011. Evaluation and long-term follow-up of infants with inborn errors of metabolism identified in an expanded screening programme. *Molecular Genetics and Metabolism* **104**: 470-475. | Duplicate (Couce 2011, No. 19) |
| Mak CM. Lee HC. Chan AY. Lam CW. 2013. Inborn errors of metabolism and expanded newborn screening: review and update. *Critical Reviews in Clinical Laboratory Sciences* **50**: 142-162. | No test accuracy data for TYR1 screening |
| Marquardt G. Currier R. McHugh DM. Gavrilov D. Magera MJ. Matern D. et al. 2012. Enhanced interpretation of newborn screening results without analyte cutoff values. *Genetics in Medicine* 14: 648-655. | No test accuracy data for TYR1 screening |
| Marsden D. Larson C. Levy HL. 2006. Newborn screening for metabolic disorders. *Journal of Pediatrics* **148**: 577-584. | No test accuracy data for TYR1 screening |
| Matern D. Tortorelli S. Oglesbee D. Gavrilov D. Rinaldo P. 2007. Reduction of the false-positive rate in newborn screening by implementation of MS/MS-based second-tier tests: the Mayo Clinic experience (2004-2007). *Journal of Inherited Metabolic Disease* **30** 585-592. | Tyrosine as primary marker and SUAC as 2nd-tier test |
| Ozben T. 2013. Expanded newborn screening and confirmatory follow-up testing for inborn errors of metabolism detected by tandem mass spectrometry. *Clinical Chemistry & Laboratory Medicine* **51**: 157-176. | No test accuracy data for TYR1 screening |
| Padilla CD. Therrell BL. 2007. Newborn screening in the Asia Pacific region. *Journal of Inherited Metabolic Disease* **30**: 490-506. | No test accuracy data for TYR1 screening |
| Pollak A. Kasper DC. 2014. Austrian Newborn Screening Program: a perspective of five decades. *Journal of Perinatal Medicine* **42**: 151-158. | No test accuracy data for TYR1 screening |
| Rinaldo P. Tortorelli S. Matern D. 2004. Recent developments and new applications of tandem mass spectrometry in newborn screening. *Current Opinion in Pediatrics* **16**: 427-433. | No test accuracy data for TYR1 screening |
| Rinaldo P. Zafari S. Tortorelli S. Matern D. 2006. Making the case for objective performance metrics in newborn screening by tandem mass spectrometry. *Mental Retardation & Developmental Disabilities Research Reviews* **12**: 255-261. | No test accuracy data for TYR1 screening |
| Roscher AA. Olgemoller B. 2004. Newborn screening for inborn errors of metabolism with tandem spectrometry in Bavaria, Germany. *LaboratoriumsMedizin* **28**: 521-524. | Screening method for TYR1 not described, only 1 FN mentioned, no other test accuracy outcomes |
| Sahai I. Zytkowicz T. Rao Kotthuri S. Lakshmi Kotthuri A. Eaton RB. Akella RR. 2011. Neonatal screening for inborn errors of metabolism using tandem mass spectrometry: experience of the pilot study in Andhra Pradesh, India. *Indian Journal of Pediatrics* **78**: 953-960. | Tyrosine as primary screening marker |
| Scolamiero, E., et al. 2015. Targeted metabolomics in the expanded newborn screening for inborn errors of metabolism. *Molecular Biosystems* **11**: 1525-1535. | No test accuracy data for TYR1 screening |
| Shi XT. Cai J. Wang YY. Tu WJ. Wang WP. Gong LM. Wang DW. Ye YT. Fang SG. Jing PW. 2012. Newborn screening for inborn errors of metabolism in mainland china: 30 years of experience. *Jimd Reports* **6**: 79-83. | No test accuracy data for TYR1 screening |
| Smon A. Murko S. Lampret BR. Battelino T. 2014. Pilot research on expanding Slovenian newborn screening programme for inherited metabolic disorder detectable by tandem mass spectrometry. *Chemicke Listy* **108**: S183-S186. | Not SUAC as primary screening marker |
| Sun A. Lam C. Wong DA. 2012. Expanded newborn screening for inborn errors of metabolism: overview and outcomes. *Advances in Pediatrics* **59**: 209-245. | No additional test accuracy data reported |
| Tarini BA. Christakis DA. Welch HG. 2006. State newborn screening in the tandem mass spectrometry era: more tests, more false-positive results. *Pediatrics* **118**: 448-456. | No test accuracy data for TYR1 screening |
| Therrell BL. Padilla CD. Loeber JG. Kneisser I. Saadallah A. Borrajo GJ. Adams J. 2015. Current status of newborn screening worldwide: 2015. *Seminars in Perinatology* **39**: 171-187. | No test accuracy data for TYR1 screening |
| Verma IC. Bijarnia S. Puri RD. 2005. "Screening for inborn errors of metabolism." *Journal of Neonatology* **19**: 107-116. | No test accuracy data of SUAC for TYR1 screening |
| Vilarinho L. Rocha H. Sousa C. Marcão A. Fonseca H. Bogas M. Osório RV. 2010. Four years of expanded newborn screening in Portugal with tandem mass spectrometry. *Journal of Inherited Metabolic Disease* **33**: S133-138. | Tyrosine as primary marker, SUAC as 2^nd^-tier test |
| Wilcken B. Haas M. Joy P. Wiley V. Bowling F. Carpenter K. Christodoulou J. Cowley D. Ellaway C. Fletcher J. Kirk EP. Lewis B. McGill J. Peters H. Pitt J. Ranieri E. Yaplito-Lee J. Boneh A. 2009. Expanded newborn screening: outcome in screened and unscreened patients at age 6 years. *Pediatrics* **124**: e241-248. | Tyrosine as screening marker |
| Yoon HR. Lee KR. Kang S. Lee DH. Yoo HW. Min WK. Cho DH. Shin SM. Kim J. Song J. Yoon HJ. Seo S. Hahn SH. 2005. Screening of newborns and high-risk group of children for inborn metabolic disorders using tandem mass spectrometry in South Korea: a three-year report. *Clinica Chimica Acta* **354**: 167-180. | Not SUAC as screening marker |

**Supplement 5.** Risk of bias and applicability concerns summary: review authors' judgements about each domain for each included study.


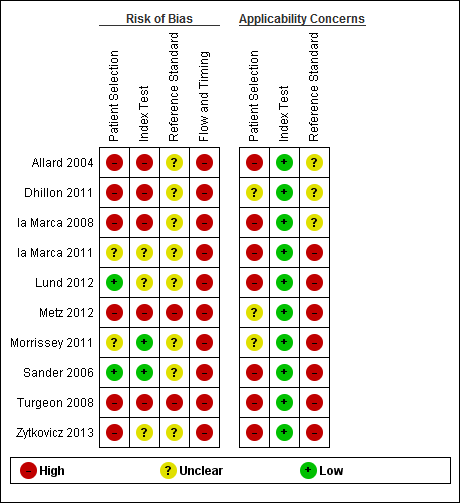


Supplement 6. Study characteristics and MS/MS screening methodology for Tyrosinemia type 1

| **Study** | **Country, time period** | **Study design** | **Source and type of material** | **Age at specimen collection** | **Samples pooled?** | **Method of extraction & derivatisation** | **Type of MS/MS conditions** | **Analyte and cut-off /**  **Levels of SUAC in affected individuals** | **Re-testing of positive screening samples /**  **Diagnostic confirmation** |
| --- | --- | --- | --- | --- | --- | --- | --- | --- | --- |
| Allard [14] | USA (Canada)  New England Newborn Screening Programme  Time period NR | Case-control study:  Stored original newborn DBS specimens of 3 known TYR1 cases and 3,199 DBS of unaffected newborns | Original newborn screening filter paper cards  Controls: stored for up to 5 days at RT;  Cases: stored at  -20°C for 4-22 months.  3.2 mm (1/8 inch) diameter filter paper disc punch | Controls: median 1.9 days;  Cases: median  2 days, range  1-3 days. | No | Extraction of SUAC from residual DBS (already extracted with methanol for AA and AC analysis) with acetonitrile: water (80:20 by volume) containing 0.1% formic acid, 15 mmol/l hydrazine hydrate (0.1% by volume), and 100 nmol/l DOA as internal standard.  Separate MS/MS analysis of SUAC- hydrazone | Quattro LC triple-quadrupole tandem mass spectrometer (Micromass Inc, USA)/  Positive ion mode;  Cone energy 20 V;  Collision energy 11eV.  SRM mode:  SUAC-hydrazone  *m/z* 155.1 → 137.1;  DOA  *m/z* 169.1 → 151.1. | Normal range SUAC  < 2 µmol/l  (study-derived) /  23.2 - 46.7 µmol/l | NR /  Clinically diagnosed cases, NR for controls |
| Dhillon [21] | California/  USA  California Newborn Blood Spot Screening Programme  1 month | Case-control study:  >1,000 NBS specimens identified as normal and stored NBS specimens of 6 confirmed TYR1 cases | 3.2 mm DBS punch | NR (newborn screening programme) | No | Simultaneous extraction of AA, AC, and SUAC from DBS using acetonitrile:water (8:2 by volume) containing 0.05% formic acid, 3.0 mmol/l hydrazine hydrate and  ^13^C_5_-SUAC as internal standard.  Derivatisation of AA, AC and SUAC-hydrazone using butanolic-HCl.  LC-MS/MS analysis of butyl esters in a single run | Triple quadrupole tandem mass spectrometer, Micromass Quattro Micro (Waters Corporation) /  Positive ion mode;  Capillary voltage 3.2 kV;  Multiple reaction monitoring;  Butyl ester of SUAC-hydrazone:  *m/z* 211.20 → 137.15;  Butyl ester of  ^13^C_5_-SUAC hydrazone  *m/z* 216.20 → 142.15 | Cut-off  SUAC 3 µmol/l  (study-derived) /  6.81 - 24.37 µmol/l | NR / NR |
| La Marca [22] | Italy  January 2007 to May 2007 | 13,000 newborn screening spots from healthy controls and 10 stored DBS samples from 6 confirmed TYR1 cases | DBS using heel stick, spotted on filter paper (903, Whatman),  3.2 mm punch (3.4 µl blood) used | Controls: 48-72 h of life;  Cases:  3 days-11 months | No | Simultaneous extraction of AA, AC and SUAC:  Addition of DOA (or ^13^C_2_-SUAC) as internal standard to the methanolic solution of deuterated AC and AA;  Extraction and derivatisation of SUAC in a single step using 3 mmol/l hydrazine in water/methanol (50:50);  Butylation;  Simultaneous MS/MS measurement of AC, AA and SUAC- hydrazone as butyl esters | Applied Biosystems/MDS Sciex API 4000^TM^ triple-quadrupole MS equipped with a TurboV-Spray® source with turbo gas temperature set at 425ºC /  Positive ionisation polarity +5500 V.  Multiple reaction monitoring:  Butyl ester of SUAC-hydrazone  *m/z* 211 → 137;  Internal standards  Butyl ester of  ^13^C_2_-SUAC-hydrazone  *m/z* 219 → 139;  Butyl ester of DOA  *m/z* 225 → 151.  Declustering enery 55V;  Collision energy 19eV | SUAC  Normal range < 2.4 µmol/l (median+5SD) / 3.3 - 18.3 µmol/l | NR / NR |
| La Marca [17] | Tuscany/  Italy  January 2007-2010 | 136,075 screened in Tuscan expanded newborn screening programme;  Overlap of 13,000 samples reported by La Marca [22] | DBS | NR  [48-72 h of life, from La Marca [22] | No | NR  (see La Marca [22] | NR  See la Marca [22] | SUAC  Normal value < 2 µmol/l /  7.6 - 14.1 µmol/l | NR /  Detection of SUAC in urine and plasma for screen-positives; NR for screen-negatives |
| Lund [18] | Denmark, Faroe Islands, Greenland  Routine expanded newborn screening programme  February 2009 to March 2011 (26 months) | Prospective routine expanded newborn screening in 140,565 newborns | DBS using heel prick, spotted om filter paper  (Schleich and Schuell 903 filter paper until 2010, then gradually replaced by the Ahlstrom 226) | 2-3 days;  Median 2.5 days.  Preterm newborns: repeated test at gestational age 32 weeks or when oral feeding had been established | No | PerkinElmer Neobase non-derivatized MS/MS kit^TM^ (3040-0010) | Waters Micromass Quattro micro™ tandem mass spectrometer / NR | SUAC > 2.1 U / NR | Flagged DBS samples re-analysed in duplicates. If the abnormal profiles were reproduced, referral to Center for Inherited Metabolic Disorders, Copenhagen University Hospital /  Urine organic acids, plasma amino acids, molecular genetic analyses for screen-positives; NR for screen-negatives |
| Metz [23] | Austria  Austrian Newborn Screening Programme 1 month | Case-control study: Prospective routine newborn screening in 4,683 consecutive newborns as healthy controls and stored DBS samples from 3 confirmed TYR1 cases | DBS (Ahlstrom 226 Paper, ID Biological, SC), one 3.2 mm punch | NR | No | MassChrom® Amino Acids and Acylcarnitines from Dried Blood; Chromsystems, Munich/Germany:  Separate SUAC extraction (including ^13^C_5_-SUAC as internal standard) from residual DBS after extraction of AA and AC;  Derivatisation solution with ~0.0005% hydrazine derived reagent;  Transfer of SUAC-hydrazone to AA and AC residues;  Butylation;  Simultaneous MS/MS measurement of AC, AA and SUAC- hydrazone as butyl esters | FIA-MS/MS analyses on a certified TQ-Detector MS system for newborn screening (Waters, Milford, MA, USA) /  Positive ion mode using a dwell time of 0.05 s.  Butyl ester of  SUAC-hydrazone  *m/z* 211 → 109.  Butyl ester of  ^13^C_5_-SUAC-hydrazone  *m/z* 216 → 114.  Cone (V): 20  Collision (eV): 24 | SUAC  Preliminary cut-off  1.29 µmol/l;  Derived from first 4,000 specimens of unaffected newborns born after 32 weeks of gestation and samples not obtained within 36 h after birth / 1.50 - 6.49 µmol/l | DBSs from potentially affected newborns were re-tested at least in duplicates from 2 separate blood spots from the same DBS card  In case of positivity, diagnostically confirmed in accordance with institutional guidelines. Unaffected newborns identified by 2^nd^ tier screening using ALAD when tyrosine > 255 µmol/l |
| Morrissey [12] | New York State/ USA  December 2007-?  (2008 and 2009, over 24 months) | ~500,000 newborns screened prospectively in New York State newborn screening programme | DBS, 3.2 mm punch from a Guthrie card (~3.1µl blood) | NR | No | Extraction of SUAC from residual DBS (after methanol extraction of AA and AC) after overnight drying using acetonitrile:water (80:20, containing 0.1% formic acid, 0.1% hydrazine, plus ^13^C_5_-SUAC as internal standard),  MS/MS analysis of SUAC hydrazone one day after AA/AC analysis | Two Waters Corp Micro LC MS/MS (Manchester, UK) with Hewlett-Packard/Agilent  Technologies series 1100 HPLC pumps.  TQD MS/MS and Acquity UPLC system (Waters Corp) for handling overload and maintenance /  Selected ion monitoring:  SUAC-hydrazone  155.1 → 137.1;  ^13^C_5_-SUAC-hydrazone  160.1 → 142.1 | SUAC  ≥ 3.00 µmol/l for retest; Average (initial and retest)  3.00-5.00 µmol/l:  repeat DBS specimen requested;  Average (initial and retest) ≥ 5.00 µmol/l:  Immediate referral /  18.29 - 19.65 µmol/l | Initial SUAC ≥ 3.00 µmol/l retested in duplicate.  Average (initial and retest) SUAC 3.00-5.00 µmol/l, repeat DBS request  Average SUAC ≥ 5.00 µmol/l immediate referral to the appropriate specialty care center./  Prenatal testing or plasma AA and SUAC with or without urine organic acids and liver function for screenpositives. NR for screen-negatives |
| Sander [19] | Germany  16 weeks | Prospective newborn screening study in 61,344 unselected newborn | DBS on S&S 903 filter paper (3.2 mm) | 36 – 72 hours after birth | No | Extraction of SUAC from residual DBS (already extracted with absolute methanol for AC and AA analysis) using acetonitrile-water (80:20 by volume) containing formic acid, 15 mmol/l hydrazine hydrate, and unlabelled DOA as internal standard.  MS/MS analysis of SUAC hydrazone in a separate run | MS/MS micro™ and Quatro LC™; Waters/Micromass Inc. / Positive ion mode, cone energy 20 V, collision voltage 10 eV,  dwell time at 9.1 s;  Multiple-reaction monitoring mode;  SUAC-hydrazone  *m/z* 155.2 → 137.1 and *m/z* 155.2 → 109.1;  DOA  *m/z* 169.3 → 151.2 | SUAC > 10 µmol/l /  152 - 271 µmol/l | NR /  Urinary SUAC and phenolic acids or tyrosine metabolites for screen-positives. NR for screen-negatives |
| Turgeon [24] | Minnesota/  USA  Mayo Clinic’s supplemental newborn screening programme  Time period NR | Case-control study: 13,521 stored random newborn screening samples not suggestive of TYR1, based on 2^nd^-tier screening and 11 stored original DBS from confirmed TYR1 | DBS | NR | No | Parallel extraction of SUAC from residual DBS (already extracted with methanol for AA and AC analysis) using acetonitrile/ water/formic acid solution (80:20:0.1, v/v/v) containing 0.1% hydrazine monohydrate (15 mmol/l) and ^13^C_5_-SUAC as internal standard.  Combined MS/MS analysis of AC and AA butyl esters, SUAC hydrazone and ^13^C_5_-SUAC hydrazone | Triple-quadrupole MS/MS (Applied Biosystems/MDS Sciex API 3000) /  Positive ion mode (source voltage, 5500 V)  Method optimisation for detection of SUAC by SRM:  SUAC-hydrazone  *m/z* 155.0 → 137.0;  ^13^C_5_-SUAC-hydrazone  *m/z* 160.0 → 142.0 | SUAC > 5.0 µmol/l /  13 - 81 µmol/l | NR /  NR for cases,  2^nd^-tier screening approach for controls |
| Zytkovicz [20] | Massachusetts/USA  New England Newborn Screening Programme  1 June 2008 to 30 June 2012  (4 years 1 month) | 518,687 samples received in New England newborn screening programme (491,472 [94.8%] born nationally; 27,215 [5.2%] born internationally) | DBS  (1/8 inch punch) | 99.4% newborn period (less than 1 month);  0.6% over 1 month of age | Yes  (up to 8 samples pooled) | Pooled sample assay:  SUAC from residual DBS (previously extracted with methanol for AA and AC) was extracted using acetonitrile: water: formic acid (80:20:0.1%) containing 17.7 mM hydrazine and 0.4 µM ^13^C_5_-SUAC as internal standard.  Up to 8 sample extracts pooled;  MS/MS analysis of SUAC hydrazone  Quantitative assay:  Untreated (newly punched) DBS were extracted and analysed as above but not pooled | Waters Quattro micro MS/MS /  SUAC-hydrazone  *m/z* 155.2 → 137.1 and  *m/z* 155.2 → 109.1;  ^13^C_5_-SUAC-hydrazone  *m/z* 160.2 → 142.1 and  *m/z* 160.2 → 114.1 | Pooled assay:  SUAC > 0.55 µM re-analysed individually /  1.1 - 3.9 µM  Quantitative assay:  SUAC  > 4 µM (recently reduced to 3.3 µM) positive;  SUAC  1.0-3.3 µM intermediate  /  4.9 - 23.1 µM | Pooled assay:  SUAC > 0.55 µM re-analysed individually  Quantitative assay:  Samples with SUAC 1.0-3.3 µM → repeat DBS specimen.  SUAC > 4 µM (recently reduced to 3.3 µM) → diagnostic testing recommended / NR (1 case had symptoms consistent with TYR1) for screen-positives. NR for screen-negatives |

AA, amino acids; AC, acylcarnitines; ALAD, 5- aminolevulinic acid dehydratase; DBS, dried blood spot; DNA, deoxyribonucleic acid; DOA, 5,7-dioxooctanoic acid; FIA, flow injection analysis; IEM, inborn errors of metabolism; LC, liquid chromatography; m/z; mass-to-charge ratio; NBS, newborn blood spot screening; NR, not reported; SUAC, succinylacetone; SRM, selected reaction monitoring; TMS, tandem mass spectrometry or tandem mass spectrometer; TYR1, Tyrosinaemia type 1
